# Investigating the Effect of an Oxytetracycline Treatment on the Gut Microbiome and Antimicrobial Resistance Gene Dynamics in Nile Tilapia (*Oreochromis niloticus*)

**DOI:** 10.3390/antibiotics10101213

**Published:** 2021-10-06

**Authors:** Christopher J. Payne, James F. Turnbull, Simon MacKenzie, Margaret Crumlish

**Affiliations:** Institute of Aquaculture, University of Stirling, Stirling FK9 4LA, UK; j.f.turnbull@stir.ac.uk (J.F.T.); simon.mackenzie@stir.ac.uk (S.M.)

**Keywords:** fish microbiome, tilapia, bacterial 16S rRNA gene, antibiotics, oxytetracycline, antibiotic resistance

## Abstract

Antibiotics play a vital role in aquaculture where they are commonly used to treat bacterial diseases. However, the impact of antibiotic treatment on the gut microbiome and the development of antimicrobial resistance in Nile tilapia (*Oreochromis niloticus*) over time remains to be fully understood. In this study, fish were fed a single treatment of oxytetracycline (100 mg/kg/day) for eight days, followed by a 14-day withdrawal period. Changes in the distal gut microbiome were measured using 16S rRNA sequencing. In addition, the abundance of antimicrobial resistance genes was quantified using real-time qPCR methods. Overall, the gut microbiome community diversity and structure of Nile tilapia was resilient to oxytetracycline treatment. However, antibiotic treatment was associated with an enrichment in *Plesiomonas*, accompanied by a decline in other bacteria taxa. Oxytetracycline treatment increased the proportion of *tetA* in the distal gut of fish and tank biofilms of the treated group. Furthermore, the abundance of *tetA* along with other tetracycline resistance genes was strongly correlated with a number of microbiome members, including *Plesiomonas*. The findings from this study demonstrate that antibiotic treatment can exert selective pressures on the gut microbiome of fish in favour of resistant populations, which may have long-term impacts on fish health.

## 1. Introduction

The aquaculture industry is a vital production sector for global food security, producing a staggering 114.5 million tonnes of aquatic protein in 2018, worth an estimated USD 263.6 billion [1] (p. 21). Within this sector, Nile tilapia (*Oreochromis niloticus*) is one of the most prevalent freshwater farmed fish species worldwide, contributing 8% of the global production in 2016 [2] (p. 23). Like many farmed fish species, the production of Nile tilapia is expected to intensify in the coming decades, in order to meet the growing demand for aquatic animal protein [3]. However, expansion of this sector is currently hampered by a number of challenges including infectious disease outbreaks from Gram-negative and Gram-positive pathogens [4,5,6], which can limit production and have huge economic impacts [7]. As a result, tilapia farmers rely on the use of antibiotics to treat or prevent bacterial diseases. Recent surveys of farmers and quantification of antibiotic residues in fish tissue have revealed the use of a diverse variety of antibiotics in tilapia farming systems from top producing countries such as Brazil, Egypt, and Vietnam [8,9,10]. These include antibiotics within the classes amphenicols, ß-lactams, fluoroquinolones, potentiated sulfonamides, and tetracyclines including oxytetracycline (OTC).

Oxytetracycline is a natural broad-spectrum antibiotic extensively used in global aquaculture due to its limited side effects in the host and broad-spectrum activity against both Gram-negative and Gram-positive bacteria, through the inhibition of protein synthesis [11,12,13]. In tilapia, OTC has been shown to successfully treat a range of bacterial diseases including Francisellosis, motile *Aeromonas* septicemia, and Streptococcosis, which are significant disease challenges for this sector [14,15,16]. Across the global aquaculture industry, OTC is often administered orally in the diet during disease outbreaks, where doses range 50 to 250 mg/kg/day for typically between four and eight days, up to 21 days, depending on the fish species and farming country [17,18]. Within the industry, a lack of understanding in antibiotic management has led to the misuse and overuse of antibiotics including OTC in production systems, with a limited understanding of how they may impact overall fish health.

The gastrointestinal tract of Nile tilapia is colonised by a community of microorganisms or “microbiome”, comprised predominantly of Fusobacteria, Firmicutes, and Proteobacteria [19,20]. In this fish species, gut microbiome members play a pivotal role in supporting host physiology and health, through microbial-mediated functions involved in disease resistance, growth, and metabolism [21,22]. Recent genomic studies have revealed this commensal community in Nile tilapia to be influenced by a number of factors commonly associated with aquaculture husbandry practices, such as alternations in salinity [23], dietary changes [22,24], and starvation [25]. Likewise, a number of studies have demonstrated that this commensal community in Nile tilapia and other fish species can also be altered following exposure to a number of antibiotics, including OTC [13,17,26,27]. However, the changes in the gut microbiome dynamics over time following OTC treatment in Nile tilapia have not been fully explored. As members within the gut microbiome serve important biological functions, understanding the short-term impacts of antibiotics on the gut microbiome is important, as any alteration or dysbiosis of this community may have detrimental effects on the physiological status of the fish host.

The continued reliance and use of antibiotics in aquaculture have contributed to the development of antimicrobial resistance (AMR) within the industry and wider environment. Indeed, high levels (>70%) of resistance have been reported to one or more antibiotic compounds in bacteria recovered from fish farms in numerous countries, including China [28], India [29], Korea [30], and Switzerland [31]. Recent genomic studies have detected numerous antibiotic resistance genes (ARGs) within the gut microbiome of freshwater carp (*Labeo rohita*) [32] and spotted sea bass (*Lateolabrax maculatus*) [33], indicating that the gut microbiome may serve as a reservoir for ARGs. Furthermore, antibiotic treatment has been demonstrated to induce selective pressures on the ARGs within the gut microbiome of fish such as Atlantic salmon (*Salmo salar*) and pacu (*Piaractus mesopotamicus*), favourably supporting the development of antibiotic resistant communities [13,34], yet little is known how antibiotic treatment influences ARG dynamics in Nile tilapia.

Due to a lack of available vaccines and other efficacious prevention tools, the tilapia sector will continue to rely heavily on the use of antibiotics to treat or prevent bacterial disease outbreaks within intensive production systems. Therefore, the objective of this study was to determine the effect of a single OTC treatment on the gut microbiome and ARG abundance in Nile tilapia over time. To achieve this, high-throughput 16S rRNA amplicon-sequencing and quantitative-PCR (qPCR) methods were applied to profile the changes in the microbiome community and the abundance of ARGs in the distal gut of fish before, at the end of antibiotic treatment, and throughout a two-week withdrawal period.

## 2. Results

### 2.1. Fish Performance

A statistically significant difference was not found in the final mean length, weight, and growth rate of fish between treatment groups and time (*p* = 0.61, *p* = 0.80, and *p* = 0.63, respectively; Table 1). However, in general, OTC-treated fish displayed lower growth rates compared with the control fish.

### 2.2. In Vitro Antimicrobial Testing of Prepared Diets

**Figure 1:**
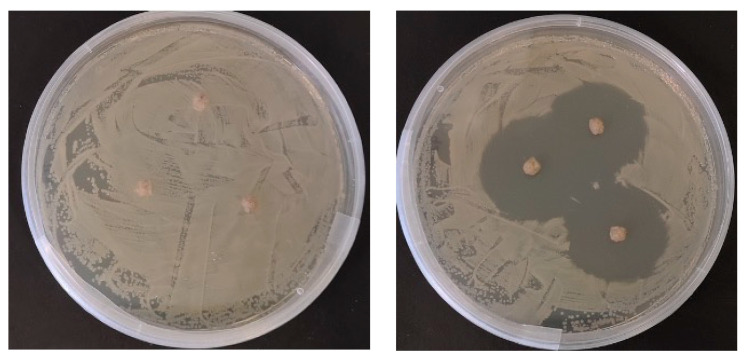
Antimicrobial activity of control and oxytetracycline-coated diets on *Aeromonas hydrophila* bacterial lawns.

Zones of inhibition in bacterial growth surrounding dietary pellets were only observed on the *Aeromonas hydrophila* bacterial lawn exposed to the OTC-coated pellets (Figure 1). These were measured at diameters of >25 mm. Control pellets that lacked OTC produced no inhibition zones after 48 h incubation, as shown in Figure 1.

### 2.3. Sequence Data and Diversity Analysis

A total of 12,733,648 reads were obtained from the Illumina MiSeq system. Following quality filtering, a total of 7,583,066 sequences remained in the final dataset and were clustered into 4450 aligned operational taxonomic units (OTUs) at a 97% similarity threshold. Of these, 2070 were observed in samples originating from fish distal gut digesta material. In addition, a total of 420, 1686, 1661, and 852 OTUs were observed in either the diet, aquarium biofilter, tank biofilms, or negative sequencing control (NSC) samples, respectively. The sequencing error was calculated at 0.0105%.

**Figure 2:**
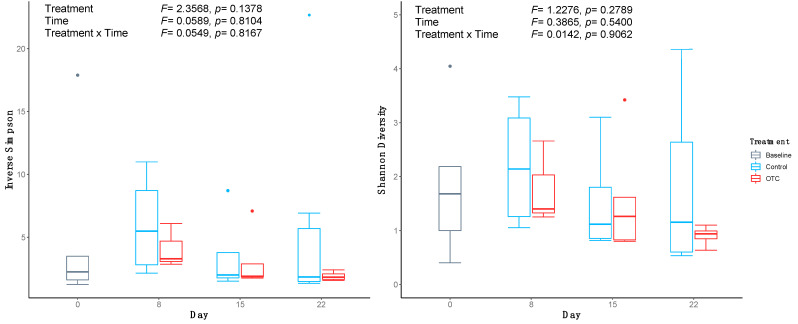
Alpha diversity measures of distal gut microbiome communities in control or oxytetracycline (OTC)-treated Nile tilapia before and after antibiotic treatment. Error bars indicate the 95% confidence interval; top, middle, and bottom of each box represent the 75th, 50th, and 25th percentiles, respectively. Circles indicate outliers from the dataset.

**Figure 3:**
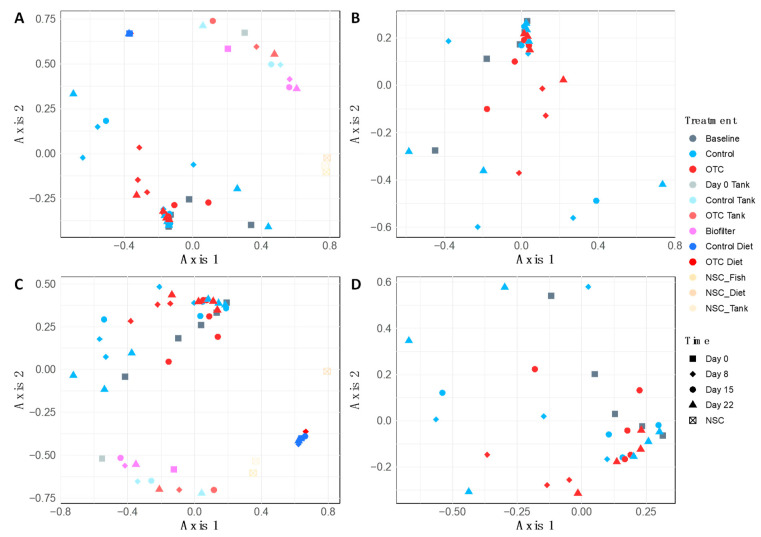
Non-multidimensional scaling of ThetaYC (**A**,**B**) and Bray−Curtis (**C**,**D**) distances. Distances illustrate differences in the microbiome community membership and composition of samples across time and exposure to oxytetracycline (OTC). Distances were generated for the complete dataset including aquarium biofilter, tank biofilm, diet, and negative sequencing control (NSC) samples (**A**,**C**), and within the distal gut of Nile tilapia alone (**B**,**D**).

Data from 31 fish across treatment groups and time points were included in the bacterial analysis following sequence quality filtering and classification. Comparing the microbial alpha diversity over time found that the distal guts of fish who consumed the OTC-coated pellets had lower microbial diversity (Inverse Simpson’s) and evenness (Shannon’s diversity), compared with the control fish group at the end of the antibiotic treatment on day 8 (Figure 2). The mean microbial evenness decreased throughout the withdrawal period in the distal gut microbiome of the OTC-treated fish, to a level below that of control fish by day 22. No significant difference was observed between the treatment groups or time. Likewise, differences in the bacterial community structure were also not statistically significant, as both the microbiome community membership and composition were found to be indistinguishable by treatment group (PERMANOVA; ThetaYC: *F* = 0.69, *p* = 0.71; Bray−Curtis: *F* = 0.59, *p* = 0.87) and time (PERMANOVA; ThetaYC: *F* = 0.66, *p* = 0.75; Bray−Curtis: *F* = 1.15, *p* = 0.30) (Figure 3B,D). Distal gut microbiome communities of fish did cluster distinctly from all other sample types including NSCs (PERMANOVA; ThetaYC: *F* = 5.15, *p* < 0.001; Bray−Curtis: *F* = 4.74, *p* < 0.001) (Figure 3A,C). As such, the taxonomic composition of the environmental and NSC samples were not investigated further.

### 2.4. Microbiome Community Dynamics in Response to OTC

**Figure 4:**
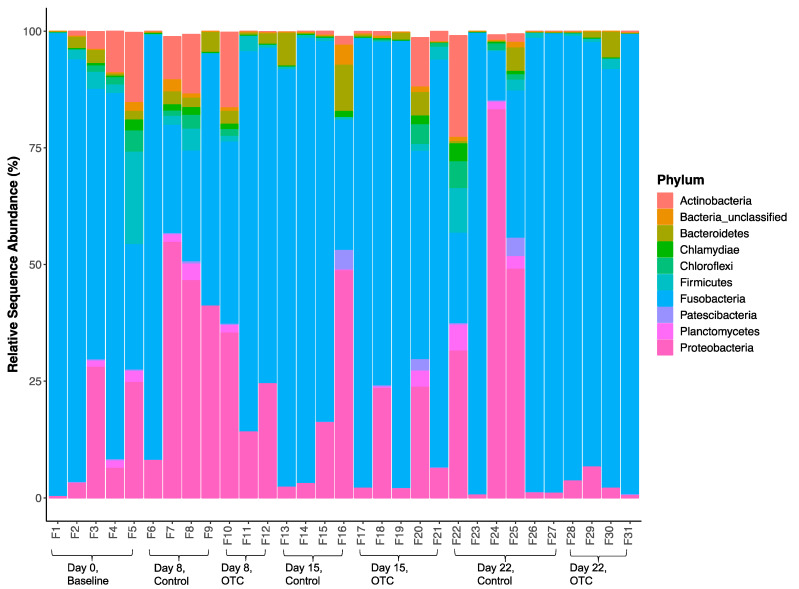
Relative sequence abundance (%) of the top 10 bacterial phyla in the distal gut of control or oxytetracycline (OTC)-treated Nile tilapia before and after antibiotic treatment.

A total of 23 bacterial phyla were observed in the distal gut of Nile tilapia, with 10 being more dominant across treatment groups and time (Figure 4). The mean (+SD) abundance of the top bacterial phyla is given in Table 2. There was considerable variation found between individual fish across treatment groups and time. Figure 4 shows that the distal gut microbiome of the baseline fish on day 0 was dominated by Fusobacteria, followed by Proteobacteria, Actinobacteria, and Bacteroidetes. The mean sequence abundance of Fusobacteria was found to increase in OTC-treated fish compared with the control fish at the end of antibiotic treatment on day 8, accompanied by a decrease in mean Proteobacteria abundance. By day 15, Actinobacteria, Chloroflexi, Firmicutes, and Planctomycetes groups were observed in higher mean abundance within the distal guts of OTC-treated fish compared with the control group. A general decline in most bacterial phyla was observed in OTC-treated fish by day 22. This shift in microbiome communities was largely driven by OTUs assigned to Fusobacteria, which was found to increase steadily in OTC-treated fish following antibiotic treatment and the withdrawal period, leading to the reduced representation of most other phyla by day 22.

**Figure 5:**
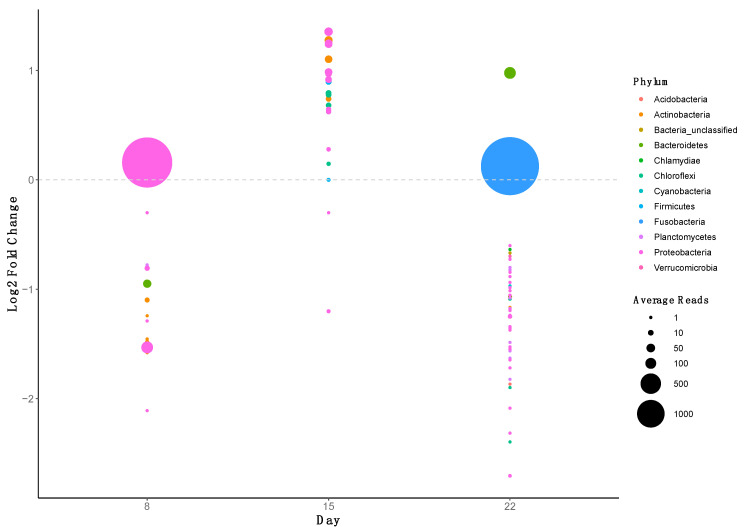
Plot of operational taxonomic units (OTU) that were significantly differentially abundant (*p* < 0.05) in the distal gut of Nile tilapia after treatment with oxytetracycline, compared with the control fish. Effect size is represented as the log2 fold-change of each OTU observed in fish from the oxytetracycline diet treatment compared with fish fed the control diet. Each circle represents a single OTU and is coloured according to the phylum to which the OTU originates. The circle size is proportional to the mean read abundance of each OTU.

In this study, LEfSe and metastats analysis were also performed to determine which OTUs were significantly different between the OTC and control groups across time (Figure 5). Further taxonomic information is given in Table S1 (Supplementary Material). Oxytetracycline treatment was associated with a statistically significant decline in the abundance of several OTUs assigned to Actinobacteria on day 8. Several Actinobacteria OTUs could not be classified to genus level, however OTC was associated with a significant decrease in a *Lamia* OTU (OTU0321) on day 8. At the genus level, OTC treatment was associated with a statistically significant decline in several Proteobacteria OTUs by day 8, assigned to *Aeromonas*, *Pseudomonas* and *Reyranella*, amongst others. In addition, sequences from both OTU0005 (*Aeromonas*) and OTU0006 (*Reyranella*), remained depleted in the distal guts of treated fish by day 22. One Proteobacteria OTU (OTU0004), assigned to *Plesiomonas*, became significantly more abundant in the distal guts of treated fish on day eight compared with the control group. At day 15, several OTUs became significantly elevated in abundance within the distal guts of treated fish. These were primarily assigned to Proteobacteria again and belonged to *Aquicella* (OTU0044) and *Hyphomicrobium* (OTU0024), amongst others. Likewise, OTUs assigned to Actinobacteria, Bacteroidetes, Chloroflexi, and Firmicutes OTUs were also found to be present at significantly higher levels, as were the Acidobacteria (OTU0065) and Chloroflexi (OTU0062) OTUs assigned to *DS-100_ge* and *RBG-13-54-9_ge*, respectively. By day 22, most differentially abundant OTUs were found to have statistically significant lower abundances in OTC-treated fish compared with control fish. These included OTUs assigned to Actinobacteria and, in particular, members belonging to *Nocardioides*, *Mycobacterium,* and a *Smaragdicoccus* OTU (OTU0063). In addition to *Aeromonas* and *Reyranella* OTUs, the abundance of several other Proteobacteria OTUs was also significantly lower on day 22 in OTC-treated fish compared with the control fish, including an OTU assigned to *Pedomicrobium* (OTU0503). Likewise, by day 22, OTUs from several other phyla also became significantly less abundant, including Chlamydiae and Verrucomicrobia, as well as several Planctomycete OTUs, assigned to *Gematta* (OTU0262 and OTU0588) and *Planctopirus* (OTU0073). This overall decline in most OTUs by day 22 was attributed to OTU0002 and OTU0020, assigned to *Cetobacterium* (Fusobacteria) and *Macellibacteroides* (Bacteroidetes), respectively, which were present at significantly higher abundances in OTC-treated fish.

### 2.5. ARG Dynamics

**Figure 6:**
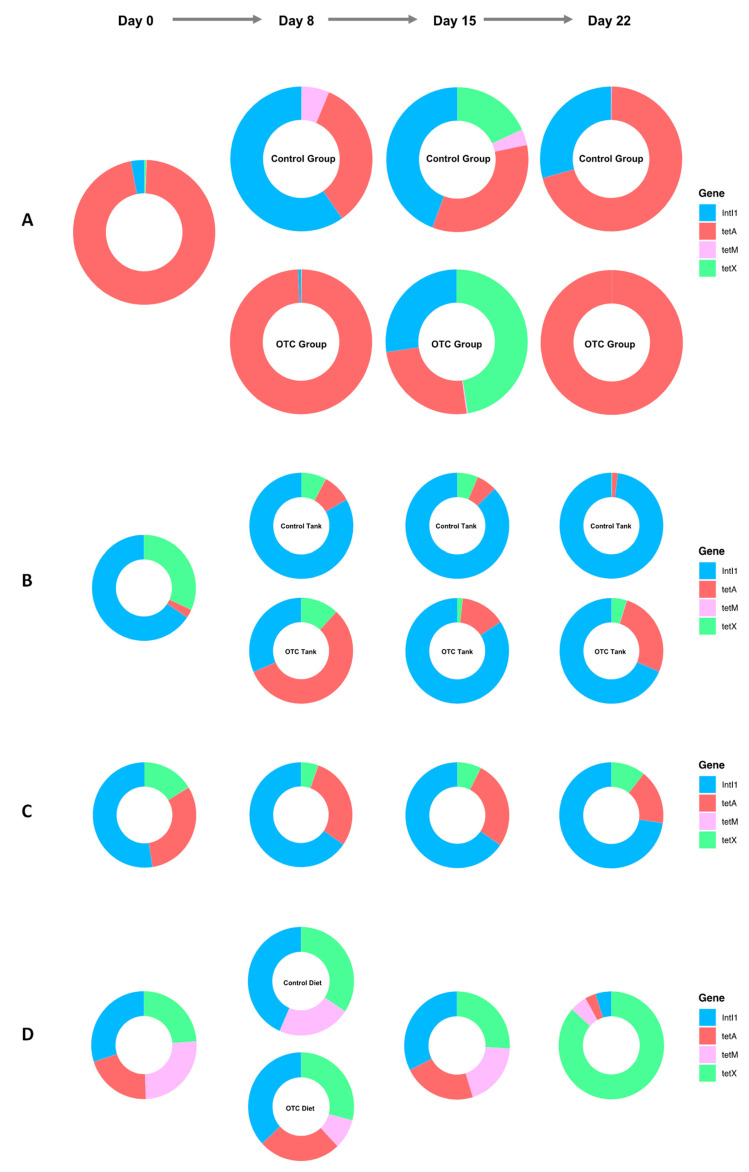
Distribution of *intI1*, *tetA*, *tetM*, and *tetX* antimicrobial resistance genes in the distal guts of fish (**A**), tank biofilms (**B**), aquarium biofilter (**C**), and diets (**D**) before and after antibiotic treatment.

*tetA* was found to dominate the distal gut of fish on day 0, followed by *intI1*, where they represented 96% and 3% of the total ARG sequences detected, respectively (Figure 6A). At the end of antibiotic treatment, *tetA* became enriched in the distal guts of the fish and in the tanks assigned to the OTC group, compared with the control group. In fact, on day 8, *tetA* made up 99% and 57%, versus 34% and 8% of the ARGs detected in OTC and control-treated fish and tank biofilms, respectively (Figure 6A,B). In contrast, *intI1* dominated the fish and tank biofilm assigned to the control group on day 8, where it represented 60% and 83%, respectively. In addition, *tetM* was also found more frequently in the control fish on day 8 compared with the OTC-treated fish, representing 6% and <0.01% of the total ARGs detected, respectively. By day 15, the prevalence of *intI1* and *tetX* increased in the OTC-treated fish, making up 27% and 48% of the total ARGs detected, respectively. Similar trends in *tetX* were also found in the control fish on day 15, where this gene represented 18% of the total ARGs detected. By day 22, *tetA* dominated (>99%) the ARGs detected within the distal guts of the OTC-treated fish. In comparison, *intI1* and *tetA* represented 29% and 71% of the ARGs detected in the distal guts of the control fish, respectively. All of the ARGs were also detected within the aquarium biofilter on day 0 (Figure 6C). Compared with the tank biofilms, the aquarium biofilter unit was more resilient to OTC treatment, as little variation was found in the distribution of ARGs over time. Likewise, all of the ARGs were also detected within most of the diet samples (Figure 6D). In addition, the distribution of ARGs detected was also fairly uniform across three out of five diet samples. More specifically, *tetA* was undetectable within the control diet on day 8, whereas *tetX* dominated the ARG profile of the diet given on day 22.

### 2.6. Correlation Analysis of ARGs and the Gut Microbiome in Fish

ARGs *intI1* and *tetM* were both positively correlated with microbiome community diversity and evenness (*p* < 0.01) (Table 3). While microbiome community evenness was positively associated with *tetX* abundance (*p* < 0.05), microbiome community diversity was only found to have weak correlations with this gene (*p* > 0.05). The abundance of *tetA* was not observed to have a strong correlation with any alpha diversity measure (*p* > 0.05).

**Figure 7:**
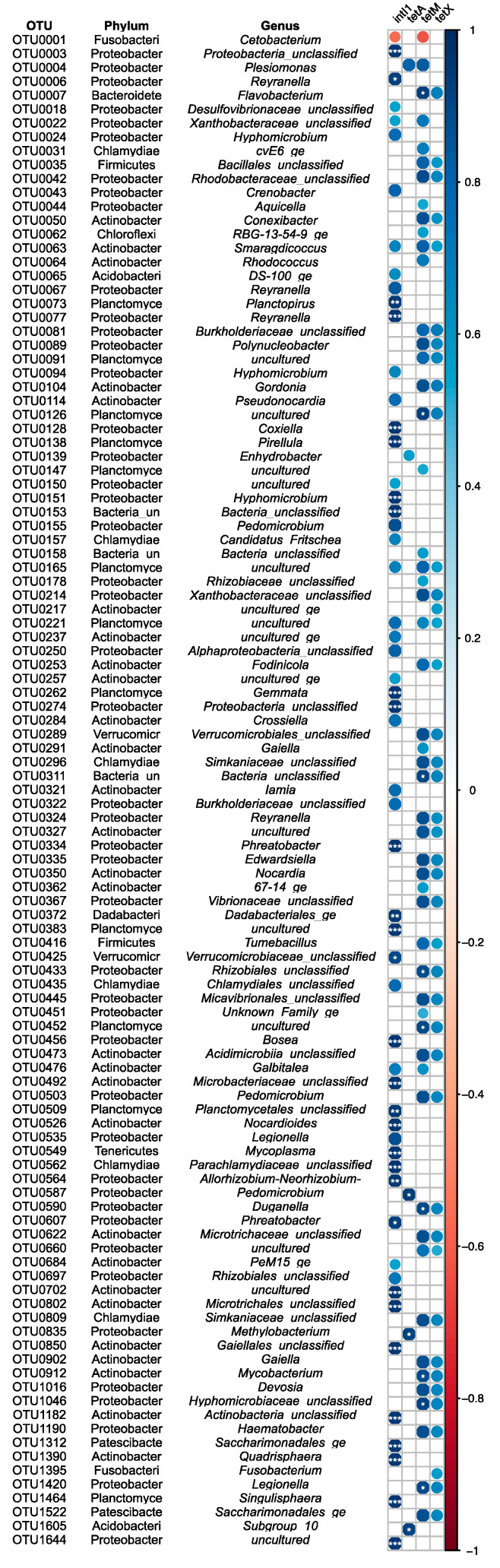
Correlation analysis between antimicrobial resistance gene abundance and operational taxonomic unit (OTU). Circle colour indicates negative or positive Pearson’s correlation coefficient according to colour scale. * denotes significance level e.g., * *p* < 0.05; ** *p* < 0.001; *** *p* < 0.0001.

A total of 109 OTUs were identified to be associated with ARG levels in the distal gut microbiome of Nile tilapia, where the magnitude of correlation ranged from −0.56 to −0.63 and 0.50 to 0.95 (Figure 7). The most diverse associations were found with *intI1*, where 55 OTUs (50.46%) had a strong positive correlation with the abundance of this gene. *tetA* was found to have the least diverse association with the microbiome community, as only five OTUs (4.58%) were found to be strongly correlated with this ARG. At the phylum level, Actinobacteria, Planctomycetes, and Proteobacteria were found to have the greatest associations with the ARGs detected, as strong correlations between these genes and the OTUs assigned to these phyla were repeatedly detected. Within the Actinobacteria phylum, 15, 14, and 12 OTUs were found to be positively associated with the abundance of *intI1*, *tetM,* and *tetX*, respectively. These OTUs were assigned to several genera, including *Mycobacterium*, *Nocardia,* and *Smaragdicoccus*. Within *Smaragdicoccus*, OTU0063 was found to be positively associated with all three ARGs. A total of eight, six, and five Planctomycetes OTUs were shown to have strong positive correlations with *intI1*, *tetM,* and *tetX* abundance, respectively. Within this phylum, most OTUs were assigned to uncultured taxa; however, OTU0262 and OTU0073 assigned to *Gemmata* and *Planctopirus*, respectively, were found to be positively associated with *intI1*. Additionally, two uncultured Planctomycete OTUs assigned to the Gemmataceae (OTU0165) and Pirellulaceae (OTU0221) families were also found to be associated with *intI1*, *tetM,* and *tetX*. A total of 23, 21, and 16 Proteobacteria OTUs were found to have positive correlations with the abundance of *intI1*, *tetM,* and *tetX*, respectively. These positively correlated OTUs were assigned to a range of genera, including *Edwardsiella* and *Legionella,* among others. Likewise, the OTUs assigned to *Aquicella* (OTU0044), *Pedomicrobium* (OTU0503), and *Reyranella* (OTU0006) were also identified as having positive associations with *tetM*, *tetX,* and *intI1*, respectively. Among the five OTUs strongly correlated with *tetA* abundance, four were classified as Proteobacteria, including OTU0004, which contributed the majority of reads assigned to *Plesiomonas*. Several other OTUs belonging to other taxonomic groups were also identified as having positive associations with ARGs. These included those assigned to *DS-100_ge* (*intI1*), *Dadabacteriales_ge* (*intI1*), *Flavobacterium* (*tetM* and *tetX*), *Mycoplasma* (*intI1*), and *Saccharimonadales_ge* (*intI1*, tetM, and *tetX*). Negative correlations were observed between the abundance of OTU0001 assigned to *Cetobacterium*, which dominated the gut microbiome communities of fish, and the ARGs *intI1* and *tetM*.

## 3. Discussion

Antibiotic treatment is a common husbandry practice used to treat and control the severity of infectious bacterial disease outbreaks in aquaculture. Moreover, this approach is of critical importance for farmed fish species like Nile tilapia, where alternative prophylactic strategies such as commercial vaccines are limited [35]. A recent culture-independent analysis revealed that antibiotic treatment can disrupt the gut microbiome, while promoting AMR within terrestrial vertebrate animals [36,37]. As members of the gut microbiome community serve vital biological functions for the fish host, understanding the intentional and unintentional consequences of antibiotic treatment on the gut microbiome community in farmed fish species is vital to support the overall health and welfare of the farmed animals.

In the study presented here, OTC at 100 mg/kg/day was not found to significantly affect overall growth performance of fish, although treated fish did have lower body weights and growth rates compared with the control group by the end of the trial. This was in agreement with findings from previous studies in Nile tilapia where fish fed OTC at 80 mg/kg/day, were not found to differ significantly in their growth performance compared with the control fish [17,38]. However, as OTC at 80 and 100 mg/kg/day has also been shown to significantly reduce and increase biomass in Nile tilapia when administered for 35 days and 12 weeks, respectively [39,40], it is likely that the lower biomass and growth rate of the treated fish compared with the control fish in the present study could become significant if the length of the treatment period was increased in future studies. Reduced growth performances in Nile tilapia treated with OTC has been previously attributed to reduced feed efficiency and nutrient digestibility through reductions in the protein and lipase enzyme activity, among other pathways [17,39]. Likewise, antibiotic-induced changes in the gut microbiome has also been implicated as an underlying mechanism in biomass changes within terrestrial vertebrate animals [41], and thus similar interactions may occur in fish.

Oxytetracycline treatment did not significantly affect bacterial diversity and evenness or community structure within the distal guts of the fish. These findings would therefore demonstrate a certain degree of resistance in the gut microbiome community of Nile tilapia in response to a single OTC exposure, and agree with a similar study in this fish species [17]. The microbiomes of both baseline and post-OTC treated fish in this study were dominated by Fusobacteria, supporting previous findings that members of this phylum are conserved in fish species, where they play a role in vitamin production for the fish host [19,25,42,43,44]. Resilience and enrichment in Fusobacteria following antibiotic treatment has been reported in zebrafish (*Danio rerio*) when exposed to OTC at similar or lower levels, respectively [45,46]. Therefore, the apparent resilience in the gut microbiome of fish in this study towards OTC could be explained by the predominance of this bacterial phyla.

Whilst the overall gut microbiome diversity and structure was resilient to OTC treatment, the abundance of numerous bacteria were found to significantly change following OTC exposure. Indeed, the most significant decrease in abundance was detected in the guts of the treated fish following OTC treatment at day 8, similar to what has been reported for this antibiotic in Atlantic salmon [13,47]. This was particularly evident for the Gram-negative Proteobacteria OTUs assigned to *Aeromonas* and *Reyranella*. Furthermore, OTUs belonging to these genera remained depleted in OTC-treated fish even after a two-week withdrawal period. A general decline in members of the Proteobacteria phylum was not surprising given that OTC is frequently used in the treatment of bacterial fish pathogens, many of which belong to Proteobacteria [48,49,50] (p. 9). However, OTC was also shown to decrease the abundance of several Gram-positive bacteria, including those assigned to *Lamia* and other Actinobacteria genera. These findings therefore demonstrate the diverse nature of organisms within the gut microbiome, which can be unintentionally targeted by OTC during antibiotic treatment on the fish farm.

The statistically significant increase in *Plesiomonas* OTU at the end of OTC treatment suggests that this genus is resistant to some antibiotic compounds, and that OTC may promote the growth of *Plesiomonas* in the fish gut. This is consistent with results from a study in pacu following treatment with florfenicol [34]. *Plesiomonas* is a Gram-negative member of the Enterobacteriaceae family, which has been previously detected within the gut microbiome of numerous fish species including discus fish (*Symphysodon haraldi*) and zebrafish [51,52]. Recently, *Plesiomonas shigelloides*, the only species within this genus, was associated with clinical disease outbreaks in fish [53], therefore results from the present study indicate that OTC treatment may promote favourable growth conditions for resistant opportunistic pathogens already established within the fish gut microbiome. This agrees with the findings from a previous study in which *Aeromonas salmonicida*, the aetiological agent of furunculosis, was found to dominate the gut of Atlantic salmon following OTC treatment [13]. In this study, OTUs were only classified to genus level, however the association found between *Plesiomonas* and OTC treatment warrants further investigation to evaluate the impact of OTC treatment and the onset of disease.

One hypothesis for the resistance of *Plesiomonas* in this study is that it could be acquired, as *tetA,* an ARG that encodes for a tetracycline efflux pump, became enriched in the distal guts of OTC-treated fish following OTC treatment. This is in agreement with previous studies that detected *tetA* in *Plesiomonas* from aquatic environments and fish including Nile tilapia [54,55,56]. Furthermore, the correlation analysis identified positive correlations between the abundances of the *Plesiomonas* OTU and the ARGs *tetA* and *tetM*, the latter of which encodes for a ribosomal protection protein. The correlation analysis also identified other positive associations between ARGs and resident microbiome members. For example, several correlations were found between the abundance of Actinobacteria members and ARGs, including a significant positive association between *Mycobacterium* and *tetM* abundances. These findings were not surprising given that the first tetracycline compounds originated from *Streptomyces aureofaciens*, another member of the Actinobacteria phylum [57]. As such, in addition to producing antimicrobial compounds, Actinobacteria members may also contain a range of mechanisms that aid in defending against their own antibiotics, as well as resistance to compounds excreted from similar organisms. In fact, *tetM* has previously been detected in several Actinobacteria genera, including *Mycobacterium* [58]. Taken together, the results from this study therefore suggest that OTC treatment may select for AMR within the gut microbiome of Nile tilapia, and supports findings from a previous study in Atlantic salmon [13]. These findings are a concern for the aquaculture industry, as the promotion of AMR within the gut microbiome from previous antibiotic exposures may reduce the effectiveness of future treatments with the same compound. This would be problematic for a number of fish farming countries, where only a small number of antibiotic compounds are licensed for use in farmed fish [50] and, as such, can be given multiple times during the production cycle.

As the fish used in this study had never previously received any antibiotic treatment, the detection of all four ARGs within the distal gut of the baseline fish was unexpected. The results could likely be explained by the colonisation of resistant microbial communities from the surrounding tank biofilm, as environmental microbiome communities are thought to colonise the developing fish gut during microbiome establishment [59]. This hypothesis is supported by the fact that the distal guts of fish and tank biofilms in this study shared several OTUs in common (data not shown). Moreover, all four ARGs investigated in this study were also detected in the tank biofilms and main biofilter unit within the aquarium. As the egg-associated microbiome community also facilitates the initial colonisation of the gut microbiome in fish [60], vertical transmission processes may have also played a role in the presence of ARGs in the baseline fish, similar to what has been reported for higher vertebrate animals [61]. In this study, the mouthbrooding behaviour of Nile tilapia may have allowed OTC-resistant bacteria carrying tetracycline ARGs within the maternal oral microbiome to transfer onto the egg surface, where they were able to colonise the distal guts of developing fish. However, to date, little is known about the vertical transmission of microbiome communities and their ARGs in fish.

This study had some limitations that should be considered when interpreting the findings. First, as non-invasive methods are currently not available or well evaluated for fish gut microbiome studies, we were unable to analyse the microbiome communities of individual fish over time. Whilst the sampling approach used in the present study is in line with other fish gut microbiome research [62,63], the development of non-terminal gut microbiome sampling methods in fish is warranted in order to improve the design of longitudinal studies. Secondly, although inter-individual variability in microbiome communities is well documented in fish and other vertebrate animals [64,65,66,67,68], the high individual variability between fish in this study may have masked any significant effects of OTC on growth performance and microbiome diversity. Whilst this study used a similar sample size to that of other fish microbiome studies [17,27,39,69], future studies would benefit from increased sample sizes so as to further explore the findings from this study. However, this number must not compromise the ethical standards of the experiment and should be in agreement with the national regulations for animal research. Finally, the analysis of microbiome communities was performed via 16S rRNA amplicon sequencing, which is reliant on deposited sequences in curated databases. Furthermore, the short read length and high sequence similarity between certain taxonomic groups also gives this method poor discriminatory power below genus level [70]. As such, to build on the findings from this study, shotgun metagenomic methods that profile whole microbial genomes are warranted to identify the taxonomic and functional changes in the microbiome at a species/strain level, as well as to detect changes in a more diverse array of ARGs following antibiotic treatment.

## 4. Materials and Methods

### 4.1. Experimental Design

The effects of OTC exposure on the distal gut microbiome and ARG abundance in Nile tilapia was performed over a 36-day time series feeding study, which took place within the aquarium facilities at the Institute of Aquaculture (IoA), University of Stirling, UK. A total of 42 mixed sex, apparently healthy Nile tilapia (mean individual weight and lengths were 48.33 ± 7.26 g and 13.69 ± 0.76 cm, respectively) were obtained from a single full-sib stock population held onsite at IoA. None of the fish had received any antibiotic treatment or vaccination prior to the start of the trial. The fish were randomly allocated into individual 19 L tanks, which were maintained on a recirculation system, at a flow rate of 1.2 L/min, under a 12:12 h light:dark cycle, and ambient water temperature of 27 ± 0.5 °C. Fish were maintained in these conditions throughout the entire trial.

Following a 14-day acclimation period, the tanks were randomly allocated into two treatment groups (n_tanks_ = 18 per treatment). Fish in treatment group one were fed a medicated diet, surface coated with OTC at 100 mg/kg/day to reflect a dose that has been reported in aquaculture [17]. Furthermore, when given at a similar or lower dose, OTC has been shown to improve growth and feed efficiency, as well as alter the gut microbiome in Nile tilapia, respectively [17,40]. Fish in treatment group two were fed a non-medicated (control) diet. Both diets were delivered into respective tanks at a rate of 1.5% bodyweight/day for eight days. After the 8-day treatment period, fish in both treatment groups were fed the control diet at a feeding rate of 1.5% bodyweight/day for 14 days, after which time the experimental trial was terminated. The feeding rate was chosen based on advice from veterinary staff and aquarium technicians following monitoring of the feeding response of fish during the acclimation period. Throughout the entire trial, diets were split into morning and afternoon rations for each fish, which were fed by hand. During each feeding period, the feeding response of individual fish was monitored to ensure the complete diet allocation was consumed.

### 4.2. Diet Preparation and In Vitro Antimicrobial Testing

The commercial pelleted feed Standard Expanded Floating Pellet 3 mm (8% oil content, 40% protein content) (Skretting, Wincham, UK) was used throughout the experiment. The OTC diet was prepared by surface coating pellets with OTC hydrochloride (98.2% purity) (Duchefa Biochemie^®^, Haarlem, The Netherlands). Following the coating of pellets with OTC, cod liver oil (Vitarenew^®^; Principle Healthcare International Limited, Skipton, UK) was applied at a rate of 20 mL/kg diet to bind the antibiotic to the pellets. The OTC diet was prepared 24 h prior to commencing the treatment period. All diets were stored at 4 °C until required.

Prior to feeding the fish, the OTC diet was tested for antimicrobial activity against the OTC-sensitive *A. hydrophila* NCIMB 9240. Briefly, a bacterial suspension was made using a colony of *A. hydrophila* inoculated into 30 mL sterile tryptone soy broth (Oxoid^®^, Basingstoke, UK) and was incubated for 18 h at 28 °C. Following incubation, the bacterial suspension was centrifuged at 2600× *g* for 15 min at 4 °C. The resulting bacterial pellet was then resuspended in sterile phosphate buffered saline (pH 7.2) to reach a MacFarland standard equivalent of 5.0, as judged by the naked eye. Then, a bacterial lawn containing a total of 100 μL of the bacterial suspension was spread onto sterile tryptone soy agar (Oxoid^®^, Basingstoke, UK). This was left for 5 min at room temperature, after which, three pellets from the OTC diet were then aseptically placed onto the agar plate. All pellets were placed carefully to ensure they remained separated. The agar plate was then sealed before incubating at 28 °C for 48 h. Bacterial growth and zones of inhibition around the diet pellets were recorded after 48 h. Pellets from the control diet were also tested to confirm they were free from any antimicrobial compounds.

### 4.3. Sample Collection

Gut digesta was aseptically collected from individual fish at four time points, which were as follows: immediately before antibiotic treatment (day 0; baseline), at the end of the antibiotic treatment (day 8), one-week post-treatment withdrawal (day 15), and at the end of the two-week withdrawal period (day 22). At each timepoint, individual fish from six tanks were randomly sampled from each treatment group, giving *n* = 6 fish per treatment group and per sampling time point. This followed the international recommendations for RNA-seq experiments [71], which use similar molecular methods, and met conditions for the 3Rs framework in animal research [72]. Following euthanasia by a lethal dose of tricaine methanesulfonate (1000 mg/g; Pharmaq^®^, Fordingbridge, UK), the fish were weighed and the total length (snout to caudal fin) recorded. Following this, the digesta from individual fish was aseptically collected from the distal portion of the gut (distal point of midgut to ~2 cm before the vent), as described by [73], except gut digesta was stored in empty 2 mL microcentrifuge tubes (Alpha Labs^®^, Eastleigh, UK). No intact feed pellets were observed within the digesta of any of the fish sampled. In addition to the gut digesta samples, a total of ten pellets from each diet (stored in sterile 7 mL containers) and biofilm samples from the main filtration unit, as well as a random tank for each treatment/time point, were also collected. Biofilm samples were collected using a sterile swab (VWR International, Monroeville, PA, USA) placed just below the water line and moved around each side of the tank/filtration unit for ca. 20 s. Biofilm samples were stored in 2 mL microcentrifuge tubes as described for the digesta samples. At each sampling point, all samples were held on ice until sampling was complete, and they were stored at −80 °C until required.

### 4.4. Library Preparation and Illumina Miseq Sequencing

A total of 162.9 ± 77.7 mg of gut digesta was processed for genomic DNA extraction, following the protocol described by [74], using the QIAamp Fast DNA Stool Mini Kit (Qiagen^®^, Manchester, UK) and 0.7 mm garnet beads (PowerBead Tubes, Qiagen^®^, Manchester, UK). The final DNA was eluted in a 35 μL EB buffer (10 mM Tris-HCl, pH 8.5; Qiagen^®^, Manchester, UK). The concentration and purity of the eluted DNA samples was measured using the Nanodrop^®^ 2000c spectrophotometer (ThermoFisher Scientific, Basingstoke, UK) and ten microlitre aliquots, and was stored at −20 °C until required. Genomic DNA was also extracted from the diet pellets (80 mg; ca. six pellets) and biofilm samples using the same commercial DNA extraction kit and method described previously. Genomic DNA was extracted for a group of NSCs in an attempt to track all sources of microbial DNA contamination in 16S rRNA libraries. No sample or DNA was added to the NSC samples, instead, the inhibitEX buffer supplied in the DNA extraction kit was used as the starting material. The NSC samples were generated for all starting material types, including the digesta (NSC_Fish), diet (NSC_Diet), and biofilm (NSC_Tank) samples, respectively. The NSC_Tank sample included a sterile swab similar to that used in the original sampling. Lastly, a mock microbiome community (IoA_MB_STD) was generated for use as an internal sequencing control. The IoA_MB_STD sample contained genomic DNA at equal concentrations from five bacterial species known to colonise fish, including *A. hydrophila* NCIMB 9240, *Edwardsiella ictaluri* NCIMB 13272, *Pseudomonas aeruginosa* ATCC 27853, *Vibrio anguillarum* NCIMB 6, and *Yersinia ruckeri* NCIMB 2194.

Prior to preparing 16S rRNA Illumina libraries, the bacterial DNA yield recovered from the gut digesta and NSC samples was quantified using TaqMan real-time qPCR methods and the primer/probe combination listed in Table 4. Real-time qPCR with absolute quantification was performed on a Stratagene Mx3005P QPCR System (Agilent Technologies LDS UK Ltd., Cheshire, UK). Quantitative analysis of the 16S rRNA gene copy number was performed in triplicate 20 μL reactions containing the following: 10 μL SensiFAST™ Probe Lo-ROX mastermix (Bioline Reagents Limited, London, UK), 0.4 μL of each forward and reverse primer (0.2 μM) (Eurofins Biomnis UK Ltd., Guildford, UK), 0.1 μL probe (0.05 μM) (Eurofins Biomnis UK Ltd., Guildford, UK), 7.1 μL nuclease-free water, and 2 μL DNA (<50 ng/μL). Duplicate no DNA template control (NTC) reactions were also included in every qPCR run to confirm qPCR reagents were free from microbial DNA contamination. Real-time qPCR conditions were as follows: an initial denaturation step at 95 °C for ten minutes, followed by 40 × cycles at 95 °C for 30 s and 60 °C for one minute. The number of 16S rRNA genes per microlitre of DNA sample was calculated from the final Ct values in each qPCR reaction using a standard curve. The standard curve was generated using plasmid DNA containing the 16S rRNA V3-4 hypervariable region insert. Briefly, plasmid DNA standards were generated using the pGEM-T Easy Vector system (Promega Corporation, Fitchburg, WI, USA) and were transformed into *Escherichia coli* XL1-Blue cells (recA1 endA1 gyrA96 thi-1 hsdR17 supE44 relA1 lac (F’ proAB lacIqZ∆M15 Tn10 [Tetr])) (Agilent Technologies Inc, Cheshire, UK), following the manufacturer’s protocol. The plasmid standards were ten-fold serially diluted to concentrations from 1 × 10^8^ to 1 × 10^3^ 16S rRNA gene copies/μL. The qPCR efficiencies and R^2^ values are detailed in Table 4.

The bacterial 16S rRNA V4 hypervariable region was amplified using the 16S_V4F and 16S_V4R cocktail primers listed in Table 4. All of the samples were amplified in triplicate 10 μL reactions using 5 μL 2X NEBNext Ultra II Q5 mastermix (New England Biolabs (UK) Ltd., Hitchin, UK), with 0.4 μL of each primer (0.2 μM) (Eurofins Biomnis UK Ltd., Guildford, UK), 0.2 μL nuclease-free water and 4 μL DNA (1.49 × 10^4^ 16S rRNA copies/μL). Amplification was conducted in a Tgradient thermal cycler (Biometra GmbH, Göttingen, Germany) under the following conditions: 98 °C for 2 min, followed by 30 × cycles of 98 °C for 15 s, 54 °C for 30 s, and 65 °C for 45 s. All PCR reactions underwent a final extension stage at 65 °C for 10 min. The generated libraries were purified using the AxyPrep Mag PCR clean up Kit (Appleton Woods Ltd., Birmingham, UK), following the manufacturer’s protocol, except with a modified 1:1 volume of library to magnetic beads. A total of 7 μL of each library was indexed using the Nextera XT index primers N7XX and S5XX (Illumina^®^, San Diego, CA, USA). Libraries were also generated for the IoA_MB_STD sample, as well as for all NSC samples. The final libraries (length ~ 381 bp) were sequenced using the Illumina MiSeq^®^ NGS system with the Illumina^®^ MiSeq Reagent Kits v2 (2 × 250 bp; 500-cycle) (Illumina^®^, San Diego, CA, USA) at IoA.

### 4.5. Bioinformatic Analysis of Illumina Miseq Data

Raw Illumina reads were demultiplexed with Casava v. 1.8 (Illumina^®^, San Diego, CA, USA), and reads representing the PhiX/internal controls or reads not matching the Illumina indices were removed. The open-source program Mothur [79] was used to process the sequence read data generated. Reads were first quality-filtered to remove sequences that contained ambiguous bases, homopolymers longer than 8 bp, and reads with sequences less than 235 bp or more than 250 bp. The reads were then further denoised, allowing for up to 2 bp differences between the duplicate sequences. The reads were assessed for chimeric sequences [80], which were then discarded before the remaining reads were aligned to the SILVA-based bacterial reference alignment [Release 132, December 2017] [81]. Any reads assigned to undesired lineages including “chloroplast”, “mitochondria”, “archaea”, “eukaryota”, or “unknown” were later discarded. The sequence reads associated with the IoA_MB_STD sample were used to calculate the sequence error rate and were then removed from the final dataset. Then, OTUs were selected using the cluster.split command and a sequence cut-off of 97%, and were classified using the SILVA database described above. Finally, singleton OTUs were removed from the final dataset. The final dataset was then rarefied to 18,145 sequences, the lowest number of sequences per sample, prior to performing any further downstream analysis. The final sample size after rarefaction was *n* = 5 for day 0, baseline; *n* = 4, *n* = 3 for the control and OTC groups at day 8, respectively; *n* = 4, *n* = 5 for the control and OTC groups at day 15, respectively; and *n* = 6, *n* = 4 for the control and OTC groups at day 22, respectively.

### 4.6. Quantification of ARGs

Real-time qPCR with absolute quantification was used to quantify the abundance of four ARGs, previously used to monitor AMR within DNA samples derived from aquatic environments [78]. Target genes included a class 1 intregrase protein (*intI1*), as well as three tetracycline resistance genes (*tetA*, *tetM,* and *tetX*) (Table 4). Quantification was performed in triplicate reactions for each DNA sample on the LightCycler^®^ 480 II platform (Roche Diagnostics Ltd, Burgess Hill, UK). The qPCR reactions were prepared to a total volume of 10 μL containing 5 μL Luminaris Color HiGreen qPCR Master Mix (ThermoFisher Scientific, Basingstoke, UK), 3 μL nuclease-free water, 0.5 μL of each forward and reverse primers (0.5 μM) (Eurofins Biomnis UK Ltd., Guildford, UK), and 1 μL DNA. The primer sequences, annealing temperatures, and expected amplicon sizes for each gene are listed in Table 4. Duplicate NTC reactions were included in every qPCR run. Quantification was performed following an initial denaturation step at 95 °C for 10 min, then 40 × cycles at 95 °C for 15 s, n °C for 30 s, and 72 °C for 30 s. Finally, a gradient of 0.11 °C per second and five reads per °C from 72 °C to 95 °C was performed for the melt-curve analysis to confirm the specificity of the amplified qPCR products. The number of gene copies per microlitre of DNA samples was calculated from the final Ct values of each reaction, using a standard curve of serially diluted plasmid containing the respective gene insert from 1 × 10^8^ to 1 × 10^1^ gene copies/μL. The plasmids were prepared in *E. coli* strain DH5α (fhuA2 Δ(argF-lacZ)U169 phoA glnV44 Φ80 Δ(lacZ)M15 gyrA96 recA1 relA1 endA1 thi-1 hsdR1) (ThermoFisher Scientific, Basingstoke, UK) using the PGEM T-Easy vector system described previously and a pool of DNA from the OTC tank biofilms at days 8, 15, and 22 as the template material. The gene copies for each sample were normalised to the 16S rRNA gene copy number of the same sample. Gene copies were used as an indicator for the relative levels of ARGs within microbiome communities before and after antibiotic treatment. The efficiencies and R^2^ values for each qPCR assay are detailed in Table 4.

### 4.7. Data Visualisation and Analysis

Data visualisation and statistical analysis were conducted in JMP^®^ version 14 and Rstudio Version 1.1.419, using ggplot2 [82], phyloseq [83], reshape2 [84], and vegan [85] packages, respectively. Differences in the final mean length, weight, and growth rate (g/day) of the fish across treatment groups and time were evaluated using two-way ANOVA. Changes in alpha diversity following antibiotic treatment were measured using the Inverse Simpson’s and the Shannon’s diversity indices, which account for species diversity as well as richness and evenness, respectively. Differences in alpha diversity measurements were also evaluated using two-way ANOVA analysis, with treatment and time as factors. However, prior to performing the analysis, all alpha diversity data were log_10_ transformed to normalise the data distribution. Distance matrices of beta diversity were generated using the thetaYC coefficient [86] and Bray–Curtis dissimilarity [87] calculators on Mothur. For all distance measures, PERMANOVA (vegan; adonis function) [88] was first used to test the differences in beta diversity according to sample type, designated as fish (distal gut), tank biofilm, aquarium biofilter, diet, or NSCs. Following this, PERMANOVA was used to further test the influence of treatment and time on the inter-sample distances of the distal gut microbiome communities. PERMANOVA was conducted using 10,000 permutations. Correlations between microbiome diversity and the abundance of ARGs were calculated using Pearson’s correlation coefficient. Furthermore, the degree of interaction and correlation between individual OTUs and ARGs was further assessed with Pearson’s correlation coefficient using the rcorr.adjust algorithm provided in the RcmdrMisc package [89]. Significance of correlations was corrected for multiple inferences using Holm’s method.

## 5. Conclusions

The findings from this study demonstrated that whilst the overall gut microbiome community of Nile tilapia remained resilient to a single treatment with OTC, the abundance of particular microbiome community members shifted. A significantly positive shift in *Plesiomonas* abundance was observed in OTC-treated fish following antibiotic treatment. Moreover, a number of Gram-negative and Gram-positive organisms significantly declined in abundance after antibiotic treatment, demonstrating the huge diversity of bacteria that can be unintentionally affected with a single application. Antibiotic treatment with OTC was also associated with an increase in the abundance of certain ARGs. Furthermore, a number of strong correlations were observed between members of the microbiome community, including *Plesiomonas*, and the abundance of several ARGs. Taken together, these findings demonstrated that antibiotic treatment does disrupt gut microbiome membership in fish, and is a potential pressure in AMR development in the recovered microbiome community. Further work is required to clarify the long-term consequences of antibiotic-induced changes in the gut microbiome and AMR development in this fish species.

## Figures and Tables

**Table 1:** Final mean (+SD) length and weight measurements for control or oxytetracycline (OTC)-treated Nile tilapia before, during, and after antibiotic treatment.

Treatment	Day	Length (cm)		Weight (g)		Growth Rate (g/day)	
		Mean	SD	Mean	SD	Mean	SD
Baseline	0	15.00	0.83	57.75	8.39	0.37	0.20
Control	8	14.63	0.95	51.88	8.29	0.33	0.37
OTC	8	14.95	1.41	54.22	14.12	0.31	0.25
Control	15	15.65	1.18	61.15	14.00	0.45	0.27
OTC	15	14.72	1.09	54.20	12.03	0.27	0.18
Control	22	15.65	1.04	62.65	12.90	0.32	0.22
OTC	22	15.17	0.71	56.37	8.42	0.28	0.18

**Table 2:** Mean (±SD) abundance of top bacterial phyla in the distal gut of control or oxytetracycline (OTC)-treated Nile tilapia before and after antibiotic treatment.

Phylum	Day 0	Day 8		Day 15		Day 22	
	Baseline	Control	OTC	Control	OTC	Control	OTC
Actinobacteria	5.78 ± 6.24	5.57 ± 6.48	5.55 ± 9.24	0.76 ± 0.83	2.89 ± 4.39	4.19 ± 8.66	0.14 ± 0.17
Bacteria_unclassified	0.48 ± 0.77	0.87 ± 1.24	0.26 ± 0.46	1.11 ± 2.11	0.34 ± 0.52	0.40 ± 0.53	0.02 ± 0.03
Bacteroidetes	1.58 ± 1.24	2.39 ± 1.74	1.95 ± 1.13	4.47 ± 4.86	1.51 ± 2.07	1.00 ± 1.96	1.84 ± 2.67
Chlamydiae	0.63 ± 0.97	0.81 ± 0.82	0.38 ± 0.66	0.33 ± 0.66	0.48 ± 0.79	0.84 ± 1.50	0.01 ± 0.01
Chloroflexi	1.62 ± 1.76	1.02 ± 1.40	0.49 ± 0.85	0.01 ± 0.01	1.06 ± 1.80	1.39 ± 2.22	0.00 ± 0.00
Firmicutes	5.53 ± 8.07	1.83 ± 20.02	1.88 ± 1.49	0.78 ± 0.18	1.00 ± 1.12	2.26 ± 3.69	1.07 ± 1.03
Fusobacteria	70.58 ± 28.91	47.98 ± 32.05	64.15 ± 22.24	73.58 ± 30.96	79.57 ± 21.57	59.44 ± 43.18	93.66 ± 4.19
Patescibacteria	0.10 ± 0.12	0.16 ± 0.20	0.08 ± 0.13	1.05 ± 2.10	0.53 ± 1.07	0.73 ± 1.58	0.00 ± 0.00
Planctomycetes	1.18 ± 1.04	1.32 ± 1.70	0.56 ± 0.96	0.06 ± 0.09	0.80 ± 1.51	1.67 ± 2.22	0.05 ± 0.09
Proteobacteria	12.46 ± 12.88	37.59 ± 20.50	24.61 ± 10.59	17.52 ± 21.68	11.47 ± 11.14	27.70 ± 33.76	3.21 ± 2.61

**Table 3:** Associations between antimicrobial resistance gene (ARG) abundance and alpha diversity measures in the distal gut microbiome of Nile tilapia.

ARG	Inverse Simpson		Shannon Diversity Index	
	R	*p*	R	*p*
*intI1*	0.58	0.0008	0.57	0.0011
*tetA*	0.2	0.3000	0.24	0.2000
*tetM*	0.69	0.0005	0.55	0.0092
*tetX*	0.21	0.3700	0.45	0.0470

R—Pearson’s correlation coefficient.

**Table 4:** Primer sets used in this study.

Primer	Target	Sequence (5′–‘3)	Size (bp)	Ta°C	Eff. (%)	R^2^	Application	Source
341F	16S rRNA (V3-4)	CCTACGGGNGGCWGCAG	464	60	114.37	0.99	16S rRNA qPCR	[75,76]
805R		GACTACHVGGGTATCTAATCC						
Probe		FAM-ATTACCGCGGCTGCTGG-MGBEQ						
16S_V4F	16S rRNA (V4)	[Illumina adapter]-AYTGGGYDTAAAGNG	245	54	N/A	N/A	Illumina Libraries	[77]
16S_V4R Cocktail		[Illumina adapter] TACCRGGGTHTCTAATCC						
		[Illumina adapter]-TACCAGAGTATCTAATTC						
		[Illumina adapter]-CTACDSRGGTMTCTAATC						
		[Illumina adapter]-TACNVGGGTATCTAATC						
intI1_F	Class 1 integrase protein	CCTCCCGCACGATGATC	280	63	101	0.99	ARG qPCR	[78]
intI1_R		TCCACGCATCGTCAGGC						
tetA_F	Tetracycline efflux pump	GCTACATCCTGCTTGCCTTC	210	64	98	0.99	ARG qPCR	[78]
tetA_R		CATAGATCGCCGTGAAGAGG						
tetM_F	Tetracycline ribosomal protection	AGTGGAGAAATCCCTGCTCGGT	149	66	107	0.99	ARG qPCR	[78]
tetM_R		TGACTATTTGGACGACGGGGCT						
tetX_F	Enzymatic modification of tetracycline	GAAAGAGACAACGACCGAGAG	131	63	99	0.99	ARG qPCR	[78]
tetX_R		ACACCCATTGGTAAGGCTAAG						

Ta; Annealing temperature. ARG; antimicrobial resistance gene.

## Data Availability

Sequence files and metadata for samples used in this study are available from the European Nucleotide Archive (ENA accession number: ERP131454).

## Supplementary Materials

The following are available online at https://www.mdpi.com/article/10.3390/antibiotics10101213/s1. Table S1: Operational taxonomic units (OTU) identified as discriminatory according to oxytetracycline exposure by Metastats and LEfSe algorithms in Mothur.
