# Supplementary material for: Investigating the Effect of an Oxytetracycline Treatment on the Gut Microbiome and Antimicrobial Resistance Gene Dynamics in Nile Tilapia (Oreochromis niloticus)
Source: Antibiotics (Basel). 2021 Oct 6;10(10):1213. doi: 10.3390/antibiotics10101213 (PMC8532870; doi:10.3390/antibiotics10101213)
Supplement: Supplementary file 1 [file antibiotics-10-01213-s001.zip › antibiotics-1382794-supplementary.pdf]

Table S1. Operational taxonomic units (OTU) identified as discriminatory according to oxytetracycline exposure by Metastats and LEfSe algorithms in Mothur.

Day	Phylotype			*p* value	
	**OTU**	**Phylum**	**Genus**	**Metastats**	**LEfSe**
**8**	Otu0140	Actinobacteria	*Kineosporiaceae_unclassified*	0.040	-
	Otu0237	Actinobacteria	*uncultured_ge*	0.027	-
	Otu0321	Actinobacteria	*Iamia*	0.049	-
	Otu0359	Actinobacteria	*Microtrichales_unclassified*	0.028	-
	Otu0802	Actinobacteria	*Microtrichales_unclassified*	0.046	-
	Otu0008	Bacteroidetes	*uncultured*	0.019	-
	Otu0308	Planctomycetes	*uncultured*	0.041	-
	Otu0004	Proteobacteria	*Plesiomonas*	0.007	0.022
	Otu0006	Proteobacteria	*Reyranella*	0.029	-
	Otu0059	Proteobacteria	*Pseudomonas*	0.027	-
	Otu0163	Proteobacteria	*Uncultured*	0.032	-
	Otu0005	Proteobacteria	*Aeromonas*	-	0.030
	Otu0017	Proteobacteria	*Deefgea*	-	0.008
	Otu0155	Proteobacteria	*Pedomicrobium*	-	0.042
	Otu0208	Proteobacteria	*Reyranella*	-	0.044
**15**	Otu0065	Acidobacteria	*DS-100_ge*	0.039	-
	Otu0010	Actinobacteria	*Nocardioides*	0.044	-
	Otu0053	Actinobacteria	*Mycobacterium*	0.037	-
	Otu0609	Actinobacteria	*Corynebacterium*	0.026	-
	Otu0028	Bacteroidetes	*Flavobacterium*	-	0.010
	Otu0062	Chloroflexi	*RBG-13-54-9_ge*	0.031	-
	Otu0219	Chloroflexi	*KD4-96_ge*	0.022	-
	Otu0434	Chloroflexi	*S085_ge*	0.046	-
	Otu0945	Chloroflexi	*JG30-KF-CM45_ge*	0.034	-
	Otu0750	Firmicutes	*Lactobacillales_unclassified*	0.028	-
	Otu2501	Firmicutes	*Bacillales_unclassified*	0.024	-
	Otu0018	Proteobacteria	*Desulfovibrionaceae_unclassified*	0.028	-
	Otu0024	Proteobacteria	*Hyphomicrobium*	0.030	-
	Otu0044	Proteobacteria	*Aquicella*	0.013	-
	Otu0051	Proteobacteria	*Rhodobacteraceae_unclassified*	0.006	-
	Otu0098	Proteobacteria	*Rhizobiales_unclassified*	0.014	-
	Otu0113	Proteobacteria	*Methyloligellaceae_unclassified*	0.048	-
	Otu0116	Proteobacteria	*Rhizobiales_unclassified*	0.004	-
	Otu0136	Proteobacteria	*Sphingomonadaceae_unclassified*	-	0.032
	Otu0169	Proteobacteria	*Pseudorhodoplanes*	0.020	-
	Otu0187	Proteobacteria	*Stenotrophomonas*	0.028	-
	Otu0196	Proteobacteria	*Rhodobacteraceae_unclassified*	0.016	-
	Otu0246	Proteobacteria	*Alphaproteobacteria_unclassified*	0.037	-

Table S1. *Continued*

Day	Phylotype			p value	
	OTU	Phylum	Genus	Metastats	LEfSe
**22**	Otu0065	Acidobacteria	*DS-100_ge*	0.028	-
	Otu0063	Actinobacteria	*Smaragdicoccus*	0.014	-
	Otu0217	Actinobacteria	*uncultured_ge*	0.042	-
	Otu0582	Actinobacteria	*Corynebacterium_1*	0.038	-
	Otu0685	Actinobacteria	*Nocardioides*	0.044	-
	Otu1031	Actinobacteria	*Mycobacterium*	0.024	-
	Otu0132	Bacteria_unclassified	*Bacteria_unclassified*	0.033	-
	Otu0505	Bacteria_unclassified	*Bacteria_unclassified*	0.029	-
	Otu0782	Bacteria_unclassified	*Bacteria_unclassified*	0.030	-
	Otu1130	Bacteria_unclassified	*Bacteria_unclassified*	0.030	-
	Otu0020	Bacteroidetes	*Macellibacteroides*	0.024	-
	Otu0435	Chlamydiae	*Chlamydiales_unclassified*	0.013	-
	Otu0544	Chlamydiae	*Chlamydia*	0.017	-
	Otu1093	Chlamydiae	*Parachlamydiaceae_unclassified*	0.040	-
	Otu0012	Chloroflexi	*uncultured*	0.029	-
	Otu0058	Chloroflexi	*JG30-KF-CM45_ge*	0.021	-
	Otu0219	Chloroflexi	*KD4-96_ge*	0.044	-
	Otu0976	Cyanobacteria	*Obscuribacterales_ge*	0.021	-
	Otu0002	Fusobacteria	*Cetobacterium*	0.004	-
	Otu0073	Planctomycetes	*Planctopirus*	0.018	-
	Otu0080	Planctomycetes	*uncultured*	0.006	-
	Otu0121	Planctomycetes	*Gemmataceae_unclassified*	0.027	-
	Otu0221	Planctomycetes	*uncultured*	0.004	-
	Otu0238	Planctomycetes	*uncultured*	0.035	-
	Otu0262	Planctomycetes	*Gemmata*	0.011	-
	Otu0478	Planctomycetes	*Pirellulaceae_unclassified*	0.011	-
	Otu0574	Planctomycetes	*uncultured*	0.003	-
	Otu0588	Planctomycetes	*Gemmata*	0.002	-
	Otu0626	Planctomycetes	*uncultured*	0.007	-
	Otu0005	Proteobacteria	*Aeromonas*	0.006	-
	Otu0006	Proteobacteria	*Reyranella*	0.013	-
	Otu0024	Proteobacteria	*Hyphomicrobium*	0.005	-
	Otu0034	Proteobacteria	*Novosphingobium*	0.045	-
	Otu0036	Proteobacteria	*Bradyrhizobium*	0.045	-
	Otu0046	Proteobacteria	*Proteobacteria_unclassified*	0.013	-
	Otu0048	Proteobacteria	*Rhodobacteraceae_unclassified*	0.043	-
	Otu0051	Proteobacteria	*Rhodobacteraceae_unclassified*	0.047	-
	Otu0116	Proteobacteria	*Rhizobiales_unclassified*	0.019	-
	Otu0150	Proteobacteria	*uncultured*	0.049	-
	Otu0189	Proteobacteria	*Xanthobacteraceae_unclassified*	0.009	-
	Otu0201	Proteobacteria	*Hyphomicrobiaceae_unclassified*	0.036	-
	Otu0204	Proteobacteria	*Hyphomicrobium*	0.042	-
	Otu0206	Proteobacteria	*Enterobacteriaceae_unclassified*	0.046	-
	Otu0229	Proteobacteria	*uncultured*	0.041	-
	Otu0258	Proteobacteria	*Arenimonas*	0.048	-
	Otu0271	Proteobacteria	*Deltaproteobacteria_unclassified*	0.011	-

Otu0314	Proteobacteria	*Xanthobacteraceae_unclassified*	0.009	-
Otu0324	Proteobacteria	*Reyranella*	0.001	-
Otu0332	Proteobacteria	*Pedomicrobium*	0.022	-
Otu0334	Proteobacteria	*Phreatobacter*	0.033	-
Otu0412	Proteobacteria	*Gammaproteobacteria_unclassified*	0.019	-
Otu0503	Proteobacteria	*Pedomicrobium*	0.030	-
Otu0594	Proteobacteria	*Rickettsiaceae_unclassified*	0.010	-
Otu0679	Proteobacteria	*Dechloromonas*	0.038	-
Otu1564	Proteobacteria	*Deltaproteobacteria_unclassified*	0.030	-
Otu0425	Verrucomicrobia	*Verrucomicrobiaceae_unclassified*	0.012	-
